# Effect of *Bacillus* sp. Supplementation Diet on Survival Rate and Microbiota Composition in Artificially Produced Eel Larvae (*Anguilla japonica*)

**DOI:** 10.3389/fmicb.2022.891070

**Published:** 2022-06-10

**Authors:** Won Je Jang, Shin-Kwon Kim, Su-Jeong Lee, Haham Kim, Yong-Woon Ryu, Min Gyu Shin, Jong Min Lee, Kyung-Bon Lee, Eun-Woo Lee

**Affiliations:** ^1^Department of Biotechnology, Pukyong National University, Busan, South Korea; ^2^Aquaculture Research Division, National Institute of Fisheries Science, Busan, South Korea; ^3^Biopharmaceutical Engineering Major, Division of Applied Bioengineering, Dong-Eui University, Busan, South Korea; ^4^Aquaculture and Applied Life Sciences Major, Division of Fisheries Life Sciences, Pukyong National University, Busan, South Korea; ^5^Department of Fisheries Biology, Pukyong National University, Busan, South Korea; ^6^Department of Biology Education College of Education, Chonnam National University, Gwangju, South Korea

**Keywords:** *Anguilla japonica*, eel, leptocephalus, survival, microbiota

## Abstract

This study was performed to investigate the effect of microbial supplementation diet on the survival rate and microbiota composition of artificially produced eel larvae. Microorganisms supplemented in the diet were isolated from wild glass eel intestines and identified as *Bacillus* sp. through 16S rRNA sequencing analysis. *In vitro* tests confirmed that the strain had no hemolytic activity and virulence genes. Microbial supplemental feeding significantly increased the survival rate of artificially produced eel larvae for 30 days post-hatchling compared with that of the control group. It also caused changes in the α-diversity, β-diversity, and relative abundance of the bacterial communities. Analysis *via* phylogenetic investigation of communities by reconstruction of unobserved states predicted that these microbial community changes would significantly increase the carbohydrate metabolism, membrane transport, and cellular community pathway of the microbial supplementation group. Therefore, microbial supplementation feeding for eel aquaculture could increase the viability of artificially produced eel larvae and alter the microbial composition to induce metabolic changes.

## Introduction

Japanese eel (*Anguilla japonica*) is a typical catadromous fish found in East Asian countries, such as Korea, Japan, China, and Taiwan (Hsu et al., 2015). Although it is considered a commercially valuable aquaculture species (Okamoto et al., 2009), eel aquaculture generally involves catching glass eels from the wild as aquaculture seeds because commercial-scale artificial breeding techniques have yet to be developed (Okamoto et al., 2009; Hsu et al., 2015). Consequently, eel aquaculture is highly dependent on the capture of glass eels. However, natural eel stock is rapidly depleting because of various factors, such as changes in the marine environment and climate, habitat destruction, and overfishing (Tsukamoto et al., 2009; Chen et al., 2014). Therefore, commercial-scale artificial propagation technology should be developed to protect natural eel resources and stabilize the aquaculture industry (Tsukamoto, 2014; Hsu et al., 2015).

Hormonally treated eels can yield consistently abundant eggs and sperm (Ohta et al., 1996a,b), which can be used for leptocephalus production and subsequent metamorphosis into glass eels (Tanaka et al., 2001, 2003). However, egg quality is still unstable (Unuma et al., 2005), and the growth and survival rates of artificially produced larvae are low (Ishikawa et al., 2001; Okamura et al., 2014; Tsukamoto, 2014). Therefore, further studies on maturation induction and larval rearing methods are needed to overcome these deficiencies (Okamoto et al., 2009).

Marine snow is composed of particulate organic matters, including dead or dying animals and phytoplankton, bacteria, and fecal matter; it can be an important food source for various organisms (Decho and Gutierrez, 2017; Shin et al., 2021). In the wild, it has also been suggested that eel larvae may feed on marine snow consistently present on the ocean surface (Otake et al., 1993; Mochioka and Iwamizu, 1996; Miller et al., 2013). They use various nutrients and substances produced by microorganisms and organic matters that constitute marine snow (Kim et al., 2018). Thus far, the most suitable feed for artificially produced eel larvae is a slurry-type feed containing shark-egg powder developed by Tanaka et al. (2001, 2003). However, it causes some problems. For instance, it is not digested and absorbed well in the intestine of the eel larvae; consequently, larvae grow slowly and have a low survival rate (Hsu et al., 2015).

Fish gut microbiota play an important role in nutrient digestion and immunological processes (Jang et al., 2021). For example, the gut microbiota of eel larvae or microbiota in marine snow may help produce monosaccharides required for the synthesis of hyaluronan, which is the main component of the bodies of preleptocephali and leptocephali (Pfeiler, 1991; Hsu et al., 2015). However, the microbiota of Japanese eel are poorly studied, and the effects of microbial supplementation on survival rate at early developmental stages are yet to be explored. Therefore, the present study is conducted to investigate the effect of a microbial supplementation diet on the survival rate and gut microbiota composition at early developmental stages of eel larvae.

## Materials and Methods

### Bacterial Isolation, Identification, and Characterization

Bacteria were isolated from the intestines of wild glass eels (5.1 ± 0.3 cm) caught in the Yeongsan River (34°46′05.2′′N 126°20′58.1′′E). Afterward, the intestines were separated, homogenized, and serially diluted with 0.85% saline solution. The suspension was spread on an LB agar plate and incubated at 25°C for 48 h. A single colony was isolated, cultured in liquid medium, and identified through 16S rRNA sequencing analysis. The isolated strain was evaluated to determine its viability at various temperatures, pH, and salinity. Hemolytic activity was examined by incubating bacteria on a blood agar base plate (Kisan Bio, South Korea) at 37°C for 48 h. Toxicity gene analysis was performed *via* PCR by using the primers shown in Supplementary Table 1. PCR was performed with a Veriti 96-Well Thermal Cycler (Applied Biosystems, Waltham, MA, United States) at Dong-Eui University Core Facility Center (Busan, South Korea).

### Experimental Diet Preparation

For the experimental feed, a slurry-type diet containing shark egg as the main raw material was used (Tanaka et al., 2001; Kim et al., 2014). Shark-egg (50 g), krill meal (6 g), Soybean peptide (3 g), fishmeal (3 g), and vitamin mix (0.3 g) were mixed at 1,200 rpm for 3 min and filtered at 90 μm before use.

### Artificial Production of Eel Larvae

Larvae were produced using a previously described method (Kagawa et al., 2005; Kim et al., 2007). Briefly, female (3 years old, 400–500 g) and male (3 years old, 300–400 g) eels were matured by administering salmon pituitary extract and human chorionic gonadotropin, respectively. Ovulation of female eels was induced with 17α, 20β-dihydroxy-4-pregnen-3-one and fertilized with artificially produced sperm.

### Rearing System and Condition for Feeding Trial

Fertilized eggs were cultured in a 1 t square tank equipped with a cylindrical net (50 cm diameter); after hatching, they were cultured in a round tank (1.5 m height and 50 cm diameter) for 6 days. A feeding trial was conducted in 6 U-shaped tanks (20 L) (Supplementary Figure 1), and each tank dispensed 500 larvae. Water temperature and flow were maintained at 23 ± 0.1°C and 0.99–1.13 L/min, respectively. Experimental feed was given five times a day (10 ml/time). The survival rate 30 days after hatching was calculated using the following equation: Survival rate (%) = number of surviving larvae/number of hatched larvae × 100.

### Microbiota Analysis

After the feeding trial, the larvae in each group were collected and washed thrice with filtered water; then, 100 larvae per group were pooled and homogenized. Bacterial DNA was isolated using FavorPrep™ Tissue Genomic DNA Extraction Mini Kit (Favorgen Biotech Corp., Taiwan) in accordance with the manufacturer’s instructions. The V3–V4 region of the isolated total DNA was amplified using primers containing the Illumina overhang adapter sequence. Afterward, library quantification, quality control, and sequencing were conducted at the Moagen (Daejeon, Republic of Korea). Data were analyzed using the EzBioCloud server.^1^

### Bioinformatics and Statistical Analysis

Normality and homogeneity of variance of all data were assessed using Shaprio–Wilk and Levene tests, respectively. Data were analyzed using IBM’s Statistical Package for the Social Sciences software (SPSS Inc., Chicago, IL, United States) following Student’s *t*-test. Statistical significance was determined at *P* < 0.05. Data were presented as means ± standard deviations (SD). Principal coordinate analysis was based on the weighted unifrac metrics of bacterial operational taxonomic units between the different diets. Linear discriminant analytical effect size (LEfSe) was applied to identify differentially displayed taxa (biomarkers) among groups with linear discriminate analysis (LDA) score > 4.0 as the threshold. PICRUSt was used to predict the functional profiling of the intestinal microflora.

## Results

### Bacterial Isolation and Identification

The 16S rRNA sequences of the isolated bacteria shared 99.86, 99.59, 99.39, and 99.32% homology with the following *Bacillus* species: *B. sonorensis* NBRC 101234^T^ (AYTN01000016), *B. haynesii* NRRL B-41327^T^ (MRBL01000076), *B. licheniformis* ATCC 14580^T^ (AE017333), and *B. paralicheniformis* KJ-16^T^ (KY694465), respectively). The isolated strain was named *Bacillus* sp. FEB-1. In general, *Bacillus* sp. FEB-1 can survive at 20–50°C, pH 6–8, and 0–4% salinity. Optimal growth conditions were 35°C and pH 7. In addition, no virulence genes (Supplementary Figure 2) and hemolytic activity based on the pathogenic strain *B. cereus* KCTC 3624 were detected

### Survival Rate and Growth of *Anguilla japonica* Larvae

Table 1 shows the survival rate, body length, and depth (vertical measurement) 30 days after hatching according to diet. The survival rate was significantly increased in the group supplemented with *Bacillus* sp. FEB-1 (BAC) compared with that of the control group (Con). However, body length and depth did not significantly differ between the two groups.

**TABLE 1:** Survival rate, total length (TL), and body depth (BD) of *Anguilla japonica* larvae.

Groups	Survival rate (%)	TL (mm)	BD (mm)	BD/TL (%)
Con	50.40 ± 2.24^a^	11.72 ± 0.74	1.13 ± 0.13	9.66 ± 0.68
BAC	66.73 ± 6.87^b^	11.51 ± 0.84	1.11 ± 0.15	9.63 ± 0.98

*Values with different superscript letters within the same column are significantly different (P < 0.05). The lack of superscript letter indicates no significant differences (P > 0.05).*

### Microbiota Analysis

The diversity estimates of the BAC group significantly differed from those of the Con group. The Shannon value of the BAC group (2.84 ± 0.11) decreased compared with that of the Con (3.60 ± 0.13) groups. The Simpson value of the BAC group (0.21 ± 0.02) increased compared with that of the Con (0.05 ± 0.01) groups. The richness estimates of the two groups did not significantly vary (Table 2).

**TABLE 2:** Alpha diversity of the bacterial communities of *A. japonica* larvae.

Groups	Richness estimate			Diversity estimate	
	ACE	CHAO	Jackknife	Shannon	Simpson
Con	730 ± 151	693 ± 140	769 ± 159	3.60 ± 0.13^b^	0.05 ± 0.01^a^
BAC	515 ± 25	487 ± 29	527 ± 36	2.84 ± 0.11^a^	0.21 ± 0.02^b^

*Values with different superscript letters within the same column are significantly different (P < 0.05). The lack of superscript letter indicates no significant differences (P > 0.05).*

**FIGURE 1:**
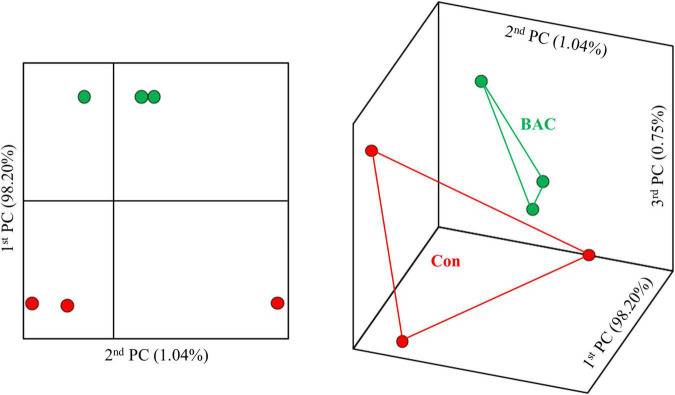
Principal coordinate analysis based on the weighted unifrac metrics of bacterial operational taxonomic units between the different diets.

Figure 1 shows the results of β-diversity analysis at the genus level based on the UniFrac metric using principal coordinate analysis. The Con and BAC groups had relatively long distances, indicating low similarity, whereas each sample in the same group had a relatively close distance. Therefore, Principal coordinate analysis elucidated clear differences between the groups.

**FIGURE 2:**
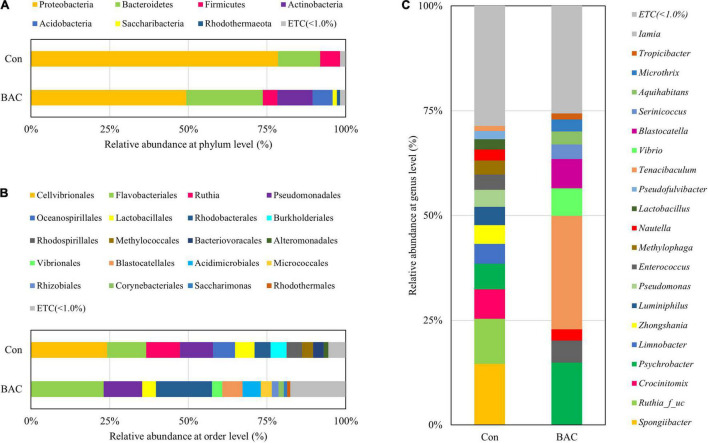
Average composition and relative abundance of bacterial communities of *Anguilla japonica* fed different diets at the phylum **(A)**, order **(B)**, and genus **(C)** levels.

The comparison of the relative abundance within the groups at the phylum level revealed that both groups were abundant in the order of *Proteobacteria* and *Bacteroidetes*. However, *Proteobacteria* accounted for a high rate of 78.48% in the Con group and a relatively low rate of 49.29% in the BAC group. The proportions of *Bacteroidetes, Actinobacteria*, and *Acidobacteria* in the BAC group were higher than those in the Con group. At the order level, *Cellvibrionales* was relatively abundant in the Con group, and *Flavobacteriales* and *Rhodobacterales* were abundant in the BAC group. At the genus level, *Spongiibacter, Ruthia* family (unclassified), and *Crocinitomix* were abundant in the Con group, and *Tenacibaculum* and *Psychrobacter* were relatively abundant in the BAC group (Figure 2).

**FIGURE 3:**
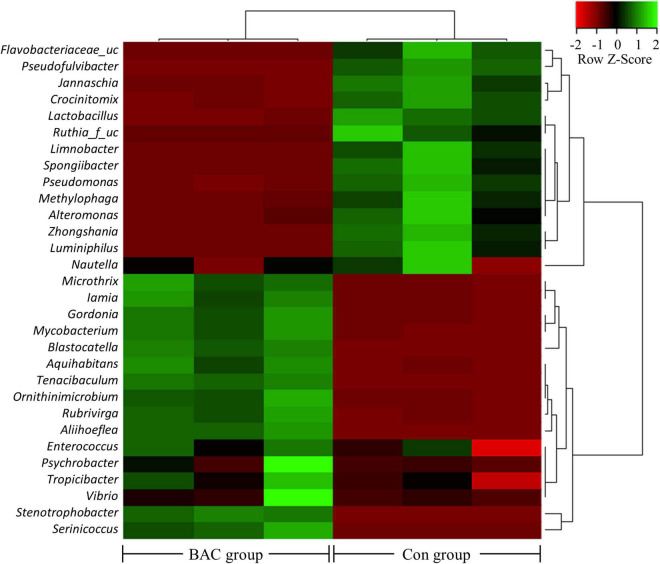
Heatmap analysis of the genus abundance within the *A. japonica* microbiota from each group. Green represents the more abundant genus in the corresponding sample and red represents the less abundant genus.

The heatmap analysis highlighting the relatively high or low levels of the top 30 selected genera is shown in Figure 3. Microbial abundance evidently differed between the two groups. In particular, 14 genera, including *Flavobacteriaceae*_uc and *Nautella*, were relatively abundant in the Con group. Conversely, 16 genera, including *Microthrix* and *Serinicoccus*, were abundant in the BAC group.

**FIGURE 4:**
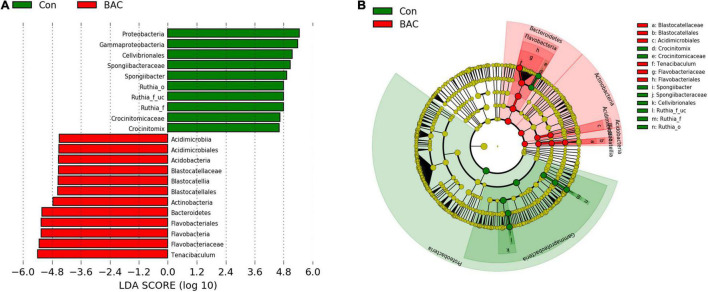
Linear discriminant analysis effect size (LEfSe) analysis of differential abundance of taxa within *A. japonica* microbiota from each group. **(A)** Linear discriminant analysis (LDA) score of abundance of taxa; **(B)** cladogram showing differentially abundant taxa between the two groups of phylum to genus.

Linear discriminant analysis effect size was used to identify significant differences in the taxa of the intestinal microbiota of *A. japonica* larvae. The Con group showed significant differences in Proteobacteria (phylum), Gammaproteobacteria (class), Cellvibrionales (order), *Spongiibacteraceae* (Family), *Spongiibacter* (genus), Ruthia (order to genus), *Crocinitomicaceae* (Family), and *Crocinitomix* (genus). the BAC group showed significant differences in the Acidimicrobiia (class), Acidimicrobiales (order), Acidobacteria (phylum), Blastocatellia (class), Blastocatellales (order), *Blastocatellaceae* (Family), Actinobacteria (phylum), Bacteroidetes (phylum), Flavobacteriales (order), Flavobacteria (class), *Flavobacteriaceae* (Family), and *Tenacibaculum* (genus) (Figure 4).

**FIGURE 5:**
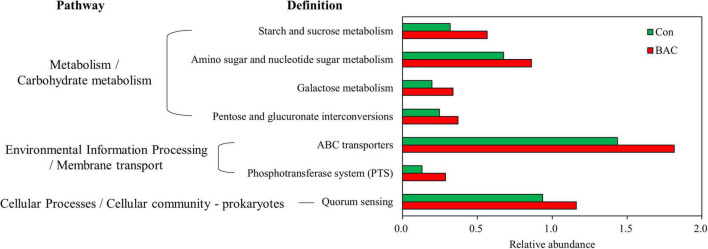
Presumptive metabolism functions of microbiota in *A. japonica* with different diets. Kyoto Encyclopedia of Genes and Genomes (KEGG) pathway was obtained from 16S metagenomic sequences using PICRUSt.

Changes in the presumptive metabolic functions of the microbiota of *A. japonica* larvae were analyzed on the basis of metagenome prediction *via* PICRUSt. The metabolic function significantly increased in the BAC group (Figure 5). The proportions of starch and sucrose metabolism, amino sugar and nucleotide sugar metabolism, galactose metabolism, pentose and glucuronate interconversions, ABC transporters, phosphotransferase system, and quorum sensing pathways in the BAC group were significantly higher than those in the Con group.

## Discussion

In this study, bacteria were isolated from the intestine of wild glass eel, and the isolated strain was identified through 16S rRNA sequencing analysis as a *Bacillus* sp. *Bacillus* spp. are abundant in fish intestines and known to provide various beneficial effects to their host (Jang et al., 2021; Santos et al., 2021). For example, they enhance digestive and antioxidant enzyme activity, immunity, and stress-related gene expression; they also produce natural antibacterial compounds that antagonize pathogens, thereby improving the ability of fish to resist pathogenic microbes (Nayak, 2010; Cha et al., 2013; Buruiană et al., 2014; Kuebutornye et al., 2019; Santos et al., 2021). With these advantages, *Bacillus* spp. can be used as probiotics in aquaculture. Many studies have reported the positive effects of *Bacillus* as probiotics on various fish species (Hasan et al., 2019; Jang et al., 2021).

Van Doan et al. (2020) defined host-associated probiotics (HAPs) as bacteria originally isolated from rearing water or the gastrointestinal tract of hosts for improving the growth and health of hosts. When used as probiotics, they may have superior functionality because they can evade their hosts’ defense system and are better adapted to the host gut environment (Van Doan et al., 2020; Jang et al., 2021). *Bacillus* sp. FEB-1 isolated from the intestines of wild glass eels is also a HAP and may provide beneficial effects on the intestines of farmed eels.

Thus far, the most suitable feed for eel larvae is a slurry-type diet based on shark-egg powder developed by Tanaka et al. (2003). Although many studies have been conducted since then, further studies on feed ingredients and composition more suitable than slurry-type diet based on shark-egg powder have yet to be performed (Kim et al., 2014). However, this slurry-type diet is not well digested and absorbed in the intestine of eel larvae; consequently, their growth is slow, and their survival rate is low (Hsu et al., 2015). Therefore, research on feed development for the advancement of artificial propagation technology on a commercial scale is important (Shin et al., 2021).

Marine snow, known as eel food in nature, contains a high amount of carbohydrates belonging to various monosaccharides and polysaccharides, such as glucose and galactose (Cowen and Holloway, 1996; Skoog et al., 2008). Saccharides are necessary for the synthesis of hyaluronan, the main component of the bodies of preleptocephali and leptocephali; they are also important for larval growth (Pfeiler, 1991; Hsu et al., 2015). Hsu et al. (2015) conducted transcriptome analysis and reported that preleptocephalus and leptocephalus stages have low transcript levels of carbohydrate-digesting enzymes. They suggested that the gut microbiota may play an important role in low nutrient digestion and immune processes in fish (Nayak, 2010; Hsu et al., 2015). They further highlighted the importance of the activity of enzymes such as glucosidase detected in bacteria isolated from marine snow (Rath and Herndl, 1994; Hsu et al., 2015). In the present study, FEB-1 supplementation likely changed the microbiota composition of eels, possibly increasing metabolism related to various carbohydrates, such as starch, sucrose, galactose, and glucuronate. These metabolic changes might have increased the survival rate of the larvae. However, further studies involving transcriptome and western blot analysis are needed to confirm whether microbial supplementation actually induces alterations in carbohydrate metabolism in eel larvae.

Other studies have used microorganisms to increase the digestibility and survival rate of larvae. Kim et al. (2018) used biofloc technology (BFT) involving *Bacillus* species. They fed larvae with biofloc similar to marine snow and investigated its effects on survival and growth. They found that the growth and survival rates of larvae fed with the BFT diet were lower than those fed with a conventional slurry-type diet. Kim et al. (2018) reported that these results may be due to differences in the composition and content of carbon compounds. Tarnecki et al. (2019) investigated the effect of *Bacillus* supplementation on common snook (*Centropomus undecimalis*) larvae. They reported that *Bacillus* supplementation can enhance the survival rate, but it cannot be accounted for the rapid larval growth (Tarnecki et al., 2019). Similarly, our result revealed that the survival rate of eel larvae increased, but their overall length did not significantly differ. Tarnecki et al. (2019) noted that *Bacillus* supplementation positively affects the fish immune system, but further studies are needed to identify this protective mechanism.

The gut microbiota composition of fish is influenced by numerous factors, such as habitat, water quality, water temperature, growth stage, and feed (Jang et al., 2021). Changes in microbiota composition can affect fish metabolism and ultimately health (Ye et al., 2011; Semova et al., 2012; Ni et al., 2014). In this study, notable changes in microbial composition at the genus level was observed in *Tenacibaculum*. For example, *T. maritimum* is a well-known pathogenic bacterium in wild and farmed marine fish (Pérez-Pascual et al., 2017). However, information about its virulence mechanisms is limited (Avendaño-Herrera et al., 2006). In this study, we could not suggest a clear association between the increased survival rate of eel larvae and increased *Tenacibaculum* in the microbiota of eel larvae by *Bacillus* supplementation. However, we found that *T. maritimum* possesses genes encoding the biosynthesis of exopolysaccharide, protease, and glycoside hydrolase (Pérez-Pascual et al., 2017). This finding suggested that these genes may influence the nutrient digestion and absorption of eel larvae.

## Conclusion

*Bacillus* sp. FEB-1 supplementation could improve the survival rate of artificially produced eel larvae and increase carbohydrate metabolism in eel larvae by changing the microbiota composition. Microbial supplementation might be used to increase survival rate in artificially produced eel larva aquaculture.

## Data Availability Statement

The original contributions presented in this study are included in the article/Supplementary Material, further inquiries can be directed to the corresponding authors.

## Author Contributions

WJJ: conceptualization, methodology, investigation, data curation, writing original draft, and formal analysis. S-KK: methodology, data curation, and funding acquisition. S-JL and HK: methodology, investigation, and data curation. Y-WR and MGS: conceptualization and funding acquisition. JML: conceptualization, methodology, data curation, and resources. K-BL: conceptualization, methodology, and writing – review and editing. E-WL: conceptualization, methodology, resources, supervision, project administration, funding acquisition, and writing – review and editing. All authors contributed to the article and approved the submitted version.

## Conflict of Interest

The authors declare that the research was conducted in the absence of any commercial or financial relationships that could be construed as a potential conflict of interest.

## Publisher’s Note

All claims expressed in this article are solely those of the authors and do not necessarily represent those of their affiliated organizations, or those of the publisher, the editors and the reviewers. Any product that may be evaluated in this article, or claim that may be made by its manufacturer, is not guaranteed or endorsed by the publisher.

## Footnotes

1
http://www.ezbiocloud.net/
