# Supplementary material for: Effect of Bacillus sp. Supplementation Diet on Survival Rate and Microbiota Composition in Artificially Produced Eel Larvae (Anguilla japonica)
Source: Front Microbiol. 2022 Jun 10;13:891070. doi: 10.3389/fmicb.2022.891070 (PMC9226642; doi:10.3389/fmicb.2022.891070)
Supplement: Supplementary file 1 [file Data_Sheet_1.DOCX]

Supplementary Material


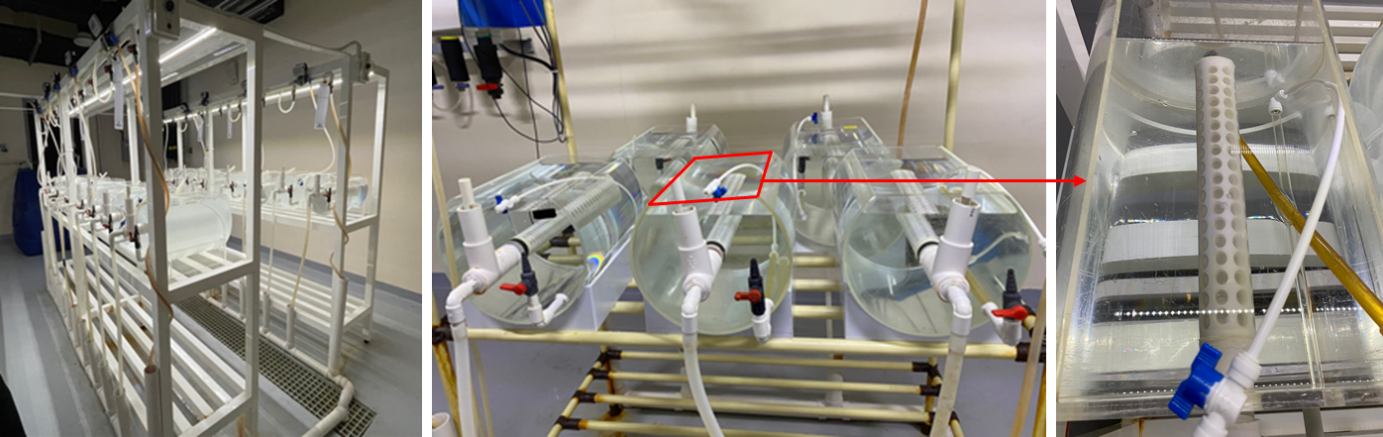


**Supplementary Figure 1.** Rearing system for feeding trial of eel larvae

**
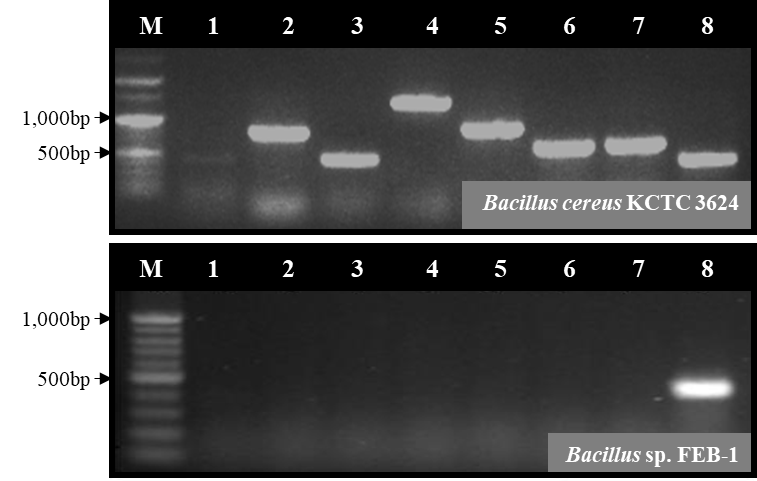
**

**Supplementary Figure 2.** PCR patterns of FEB-1 and *B*. *cereus* screened for toxigenic genes. Lane M. DNA ladder; Lane 1. hemolysin; Lane 2. nonhemolytic enterotoxin; Lane 3. enterotoxin T; Lane 4. enterotoxin FM; Lane 5. cytotoxin K; Lane 6. sphingomyelinase; Lane 7. phospholipase; Lane 8. positive control

| **Supplementary Table 1.** Gene specific primers used to amplify target genes encoding virulence factors | | | | |
| --- | --- | --- | --- | --- |
| Gene | Product | Sense | Oligonucleotide Sequence (5` to 3`) | Size (bp) |
| *Hbl* | Hemolysin | F | GGAGCGGTCGTTATTGTTGT | 620 |
|  |  | R | GCCGTATCTCCATTGTTCGT |  |
| *NheB* | Non-hemolytic enterotoxin | F | CTATCAGCACTTATGGCAG | 770 |
|  |  | R | ACTCCTAGCGGTGTTCC |  |
| *BceT* | Enterotoxin T | F | TTACATTACCAGGACGTGCTT | 430 |
|  |  | R | TGTTTGTGATTGTAATTCAGG |  |
| *EntFM* | Enterotoxin FM | F | ATGAAAAAAGTAATTTGCAGG | 1270 |
|  |  | R | TTAGTATGCTTTTGTGTAACC |  |
| *CytK* | Cytotoxin K | F | ACAGATATCGGKCAAAATGC | 810 |
|  |  | R | TCCAACCCAGTTWSCAGTTC |  |
| *Sph* | Sphingomyelinase | F | CGTGCCGATTTAATTGGGGC | 560 |
|  |  | R | CAATGTTTTAAACATGGATGCG |  |
| *piplc* | Phospholipase | F | CGCTATCAATGGACCATGG | 570 |
|  |  | R | GGACTATTCCATGCTGTACC |  |
